# *catena*-[[(nitrato-κ*O*)silver(I)]-μ-1,10-phenanthroline-5,6-dione-κ^4^ *O*,*O*′:*N*,*N*′]

**DOI:** 10.1107/S1600536811020939

**Published:** 2011-06-18

**Authors:** Xiao Jing, Yu-Lan Zhu, Kui-Rong Ma, Li Cao, Shuai Shao

**Affiliations:** a Department of Chemistry, Northeast Normal University, Changchun 130021, People’s Republic of China; b Jiangsu Key Laboratory for the Chemistry of Low-Dimensional Materials, Huaiyin Normal University, Huaian 223300, People’s Republic of China

## Abstract

In the title one-dimensional coordination polymer, [Ag(NO_3_)(C_12_H_6_N_2_O_2_)]_*n*_, the Ag^I^ atom is penta­coordinated by two N atoms from a 1,10-phenanthroline-5,6-dione (phen-dione) ligand, one O atom from the nitrate anion and two O atoms from another phen-dione ligand. The coordination environment around silver is slightly distorted square-pyramidal. Inter­estingly, the Ag—O distances to the phen-dione ligand are different [Ag—O = 2.612 (6) and 2.470 (5) Å]. The one-dimensional chains run parallel to [101] and are further inter­connected by weak hydrogen bonds (C—H⋯O) and π–π stacking inter­actions [centroid–centroid distances 3.950 (4) and 3.792 (4) Å], forming a three-dimensional supra­molecular network.

## Related literature

For the use of 1,10-phenanthroline-5,6-dione (phen-dione) as an efficient chelating ligand establishing coordination polymers, see: Calderazzo *et al.* (2002 ▶); Wu *et al.* (1996 ▶); Liu & Xu (2006 ▶); Li *et al.* (2005 ▶). For examples of complexes with *N*,*O*-coordination of phen-dione, see: Paw & Eisenberg (1997 ▶); Ruiz *et al.* (1999 ▶); Shavaleev *et al.* (2003 ▶). For the synthesis of phen-dione, see: Paw & Eisenberg (1997 ▶). For the structure of a related phen-dione complex of Ag^I^, see: Onuegbu *et al.* (2009 ▶). For a comparison of Ag—O bond lengths, see: Young & Hanton (2008 ▶); Sun *et al.* (2010 ▶); Wang *et al.* (2011 ▶).
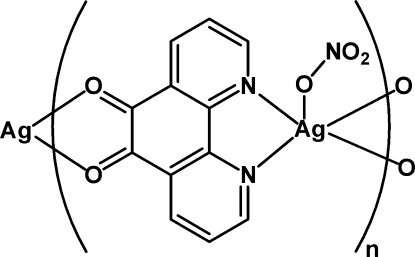


## Experimental

### 

#### Crystal data

[Ag(NO_3_)(C_12_H_6_N_2_O_2_)]
*M* *_r_* = 380.07
Monoclinic, 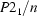

*a* = 9.5058 (14) Å
*b* = 10.4647 (15) Å
*c* = 12.1615 (17) Å
β = 99.766 (2)°
*V* = 1192.2 (3) Å^3^
*Z* = 4
Mo *K*α radiation
μ = 1.72 mm^−1^
*T* = 296 K
0.3 × 0.2 × 0.1 mm

#### Data collection

Bruker APEXII CCD area-detector diffractometer
Absorption correction: multi-scan (*SADABS*; Bruker, 2000 ▶) *T*_min_ = 0.66, *T*_max_ = 0.84
6626 measured reflections
2307 independent reflections
1374 reflections with *I* > 2σ(*I*)
*R*_int_ = 0.041

#### Refinement

*R*[*F* ^2^ > 2σ(*F* ^2^)] = 0.052
*wR*(*F* ^2^) = 0.124
*S* = 1.00
2307 reflections
190 parameters
32 restraints
H-atom parameters constrained
Δρ_max_ = 0.84 e Å^−3^
Δρ_min_ = −1.05 e Å^−3^

### 

Data collection: *APEX2* (Bruker, 2004 ▶); cell refinement: *SAINT* (Bruker, 2004 ▶); data reduction: *SAINT*; program(s) used to solve structure: *SHELXS97* (Sheldrick, 2008 ▶); program(s) used to refine structure: *SHELXL97* (Sheldrick, 2008 ▶); molecular graphics: *ORTEP-3 for Windows* (Farrugia, 1997 ▶); software used to prepare material for publication: *SHELXL97* and *PLATON* (Spek, 2009 ▶).

## Supplementary Material

Crystal structure: contains datablock(s) I, global. DOI: 10.1107/S1600536811020939/im2282sup1.cif

Structure factors: contains datablock(s) I. DOI: 10.1107/S1600536811020939/im2282Isup2.hkl

Additional supplementary materials:  crystallographic information; 3D view; checkCIF report

## Figures and Tables

**Table 1:** Hydrogen-bond geometry (Å, °)

*D*—H⋯*A*	*D*—H	H⋯*A*	*D*⋯*A*	*D*—H⋯*A*
C1—H1⋯O4^i^	0.93	2.37	3.21 (1)	149

Symmetry code: (i) 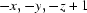.

**Fig. 1.**
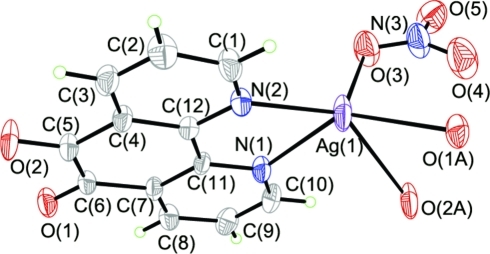
Molecular structure of the title compound, displacement ellipsoids are drawn at the30% probability level.

**Fig. 2.**
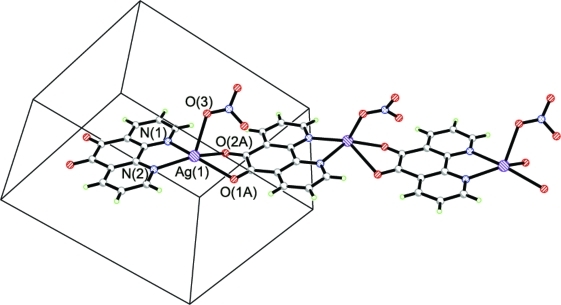
One-dimensional polymeric structure of the title compound.

**Fig. 3.**
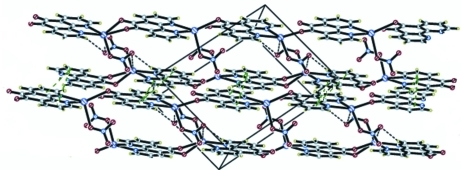
Crystal packing of the title compound, π–π stacking interactions are shown as green dashed lines and hydrogen bonds are shown as black dashed lines.

## supplementary crystallographic information

### Comment

1, 10-phenanthroline-5,6-dione (phen-dione) is an efficient chelating ligand exhibiting two types of coordinating atoms (N and O) that are electronically coupled due to conjugation throughout the ligand. While phen-dione usually binds to metals through N atoms, in some cases both the N and O atoms are used simultaneously (Paw & Eisenberg,1997; Ruiz *et al.*, 1999; Shavaleev *et al.*, 2003).

In this paper we report the synthesis and characterization of the title compound, [Ag(phen-dione)(NO_3_)]_n_ (1). Single-crystal X-ray diffraction study of 1 reveals the asymmetric unit to consist of one Ag (I) ion, one phen-dione ligand and one nitrate anion (Fig. 1). The silver atom is coordinated to two N atoms of a 1,10-phenanthroline-5,6-dione (phen-dione), one O atom from the nitrate anion and two O atoms from another phen-dione ligand giving rise to a slightly distorted square-pyramidal coordination sphere. The corresponding Ag–N bond distances are 2.445 (6) Å for Ag–N(1), 2.287 (6) Å for Ag–N(2) and Ag–O bond distances are observed to 2.474 (6) Å for Ag–O(1 A), 2.438 (7) Å for Ag–O(3) and 2.612 (6) Å for Ag–O(2 A). The latter bond length is longer than Ag–O(1 A) and Ag–O(3), but is well–matched to other examples reported in the literature (Young & Hanton, 2008; Wang *et al.*, 2011; Sun *et al.*, 2010).

In compound 1, the basic building units with a [AgN_2_O_3_]center are firstly interconnected to each other to produce a one-dimensional chain by additional Ag—O(1 A) and Ag—O(2 A) bonds (Fig. 2). Then the adjacent one-dimensional chains are further linked to each other to form three-dimensional network structure by the weak hydrogen bond C (1)–H(1)···O(4) (3.184 Å) between a CH function of a pyridine and an oxygen atom of the nitrate anion and π–π stacking interactions (Fig. 3). Two types of aromatic stacking interactions in 1 are observed between pyridine ring I (N (1), C (10), C (9), C (8), C (7), C (11)) and pyridine ring II (N (1), C (10), C (9), C (8), C (7), C (11)). There is one close distance between the centroids of pyridine I and II and another between two neighboring pyridine I moieties. The respective centroid-to-centroid distances are 3.950 (4) Å and 3.792 (4) Å.

The combination of coordinative bonds, hydrogen bonds, and π–π stacking interactions assemble the one-dimensional chain into a complicated three-dimensional supramolecular network.

### Experimental

The title compound was synthesized according to the following steps: a solution of silver (I) nitrate (0.068 g, 0.4 mmol) and phen-dione (0.0212 g, 0.1 mmol) in methanol (8 ml) was stirred for 2 h. After filtering, the filtrate was left at room temperature for about two weeks. Yellow block shaped crystals of 1 were collected by vacuum filtration, washed thoroughly with methanol dried in air (yield 23% based on silver).

### Refinement

All non-hydrogen atoms were located from the difference Fourier maps, and were refined anisotropically. All H atoms were positioned geometrically, and were allowed to ride on their corresponding parent atoms with *U*iso = 1.2 *U*eq.

### Crystal data

**Table:**

[Ag(NO_3_)(C_12_H_6_N_2_O_2_)]	*F*(000) = 744
*M**_r_* = 380.07	*D*_x_ = 2.117 Mg m^−^^3^
Monoclinic, *P*2_1_/*n*	Mo *K*α radiation, λ = 0.71073 Å
Hall symbol: -P 2yn	Cell parameters from 1252 reflections
*a* = 9.5058 (14) Å	θ = 2.6–22.6°
*b* = 10.4647 (15) Å	µ = 1.72 mm^−^^1^
*c* = 12.1615 (17) Å	*T* = 296 K
β = 99.766 (2)°	Block, yellow
*V* = 1192.2 (3) Å^3^	0.3 × 0.2 × 0.1 mm
*Z* = 4	

### Data collection

**Table:**

Bruker APEXII CCD area-detector diffractometer	2307 independent reflections
Radiation source: fine-focus sealed tube	1374 reflections with *I* > 2σ(*I*)
graphite	*R*_int_ = 0.041
Detector resolution: 0 pixels mm^-1^	θ_max_ = 26.0°, θ_min_ = 2.6°
φ and ω scans	*h* = −11→11
Absorption correction: multi-scan (*SADABS*; Bruker 2000)	*k* = −12→12
*T*_min_ = 0.66, *T*_max_ = 0.84	*l* = −14→7
6626 measured reflections	

### Refinement

**Table:**

Refinement on *F*^2^	Primary atom site location: structure-invariant direct methods
Least-squares matrix: full	Secondary atom site location: difference Fourier map
*R*[*F*^2^ > 2σ(*F*^2^)] = 0.052	Hydrogen site location: inferred from neighbouring sites
*wR*(*F*^2^) = 0.124	H-atom parameters constrained
*S* = 1.00	*w* = 1/[σ^2^(*F*_o_^2^) + (0.0445*P*)^2^ + 3.5057*P*] where *P* = (*F*_o_^2^ + 2*F*_c_^2^)/3
2307 reflections	(Δ/σ)_max_ < 0.001
190 parameters	Δρ_max_ = 0.84 e Å^−^^3^
32 restraints	Δρ_min_ = −1.05 e Å^−^^3^

### Special details

**Table:**

Geometry. All e.s.d.'s (except the e.s.d. in the dihedral angle between two l.s. planes) are estimated using the full covariance matrix. The cell e.s.d.'s are taken into account individually in the estimation of e.s.d.'s in distances, angles and torsion angles; correlations between e.s.d.'s in cell parameters are only used when they are defined by crystal symmetry. An approximate (isotropic) treatment of cell e.s.d.'s is used for estimating e.s.d.'s involving l.s. planes.
Refinement. Refinement of *F*^2^ against ALL reflections. The weighted *R*-factor *wR* and goodness of fit *S* are based on *F*^2^, conventional *R*-factors *R* are based on *F*, with *F* set to zero for negative *F*^2^. The threshold expression of *F*^2^ > σ(*F*^2^) is used only for calculating *R*-factors(gt) *etc*. and is not relevant to the choice of reflections for refinement. *R*-factors based on *F*^2^ are statistically about twice as large as those based on *F*, and *R*- factors based on ALL data will be even larger.

### Fractional atomic coordinates and isotropic or equivalent isotropic displacement parameters (Å^2^)

**Table:**

	*x*	*y*	*z*	*U*_iso_*/*U*_eq_	
Ag1	0.20025 (7)	0.15378 (7)	0.49084 (6)	0.0703 (3)	
C1	0.3824 (8)	−0.0492 (8)	0.3745 (6)	0.055 (2)	
H1	0.3348	−0.1065	0.4136	0.066*	
C2	0.4710 (9)	−0.0969 (8)	0.3058 (7)	0.060 (2)	
H2	0.4815	−0.1846	0.2981	0.072*	
C3	0.5436 (8)	−0.0134 (7)	0.2488 (6)	0.0489 (19)	
H3	0.6034	−0.0437	0.2015	0.059*	
C4	0.5264 (7)	0.1159 (6)	0.2628 (6)	0.0399 (17)	
C5	0.6048 (7)	0.2087 (6)	0.2031 (6)	0.0449 (18)	
C6	0.5831 (7)	0.3489 (6)	0.2189 (5)	0.0416 (16)	
C7	0.4826 (7)	0.3887 (7)	0.2938 (5)	0.0391 (17)	
C8	0.4591 (7)	0.5165 (7)	0.3107 (6)	0.0485 (19)	
H8	0.5058	0.5787	0.2758	0.058*	
C9	0.3654 (8)	0.5503 (8)	0.3800 (6)	0.053 (2)	
H9	0.3460	0.6358	0.3918	0.064*	
C10	0.3008 (8)	0.4552 (8)	0.4318 (6)	0.056 (2)	
H10	0.2393	0.4790	0.4801	0.067*	
C11	0.4114 (7)	0.2975 (6)	0.3479 (5)	0.0356 (16)	
C12	0.4337 (7)	0.1596 (7)	0.3331 (5)	0.0380 (15)	
N1	0.3214 (6)	0.3316 (6)	0.4165 (5)	0.0439 (14)	
N2	0.3618 (6)	0.0775 (6)	0.3874 (5)	0.0424 (14)	
N3	−0.1319 (8)	0.2293 (8)	0.4504 (7)	0.0784 (15)	
O1	0.6449 (5)	0.4259 (5)	0.1699 (4)	0.0567 (14)	
O2	0.6867 (6)	0.1721 (5)	0.1428 (5)	0.0708 (17)	
O3	−0.0348 (6)	0.2265 (7)	0.3995 (6)	0.0846 (15)	
O4	−0.1158 (8)	0.1860 (9)	0.5450 (7)	0.126 (2)	
O5	−0.2488 (6)	0.2729 (7)	0.4152 (5)	0.0896 (18)	

### Atomic displacement parameters (Å^2^)

**Table:**

	*U*^11^	*U*^22^	*U*^33^	*U*^12^	*U*^13^	*U*^23^
Ag1	0.0779 (5)	0.0783 (5)	0.0691 (5)	0.0005 (4)	0.0539 (4)	−0.0004 (4)
C1	0.062 (5)	0.052 (5)	0.057 (5)	−0.006 (4)	0.027 (4)	0.012 (4)
C2	0.066 (5)	0.047 (5)	0.071 (6)	0.003 (4)	0.023 (5)	0.004 (4)
C3	0.048 (4)	0.052 (5)	0.051 (5)	0.008 (4)	0.017 (4)	−0.006 (4)
C4	0.040 (4)	0.042 (4)	0.040 (4)	0.007 (3)	0.016 (3)	−0.004 (3)
C5	0.047 (4)	0.052 (5)	0.041 (4)	0.001 (3)	0.022 (4)	0.001 (3)
C6	0.040 (4)	0.053 (4)	0.035 (4)	−0.006 (4)	0.015 (3)	0.002 (4)
C7	0.040 (4)	0.047 (4)	0.034 (4)	−0.003 (3)	0.016 (3)	0.001 (3)
C8	0.053 (4)	0.046 (5)	0.051 (5)	−0.002 (4)	0.020 (4)	0.004 (4)
C9	0.069 (5)	0.042 (4)	0.053 (5)	0.009 (4)	0.019 (4)	−0.004 (4)
C10	0.062 (5)	0.064 (6)	0.048 (5)	0.012 (4)	0.027 (4)	−0.002 (4)
C11	0.038 (4)	0.042 (4)	0.029 (4)	0.005 (3)	0.013 (3)	−0.004 (3)
C12	0.036 (3)	0.045 (4)	0.035 (4)	−0.003 (3)	0.011 (3)	−0.003 (3)
N1	0.045 (3)	0.045 (4)	0.047 (4)	0.003 (3)	0.024 (3)	−0.002 (3)
N2	0.044 (3)	0.042 (4)	0.045 (3)	0.001 (3)	0.019 (3)	0.003 (3)
N3	0.054 (3)	0.111 (4)	0.077 (3)	0.003 (3)	0.029 (3)	0.021 (3)
O1	0.059 (3)	0.058 (3)	0.062 (3)	−0.008 (3)	0.034 (3)	0.002 (3)
O2	0.088 (4)	0.066 (4)	0.075 (4)	0.012 (3)	0.062 (3)	0.003 (3)
O3	0.061 (3)	0.108 (4)	0.092 (3)	0.006 (3)	0.036 (3)	0.009 (3)
O4	0.095 (4)	0.188 (5)	0.092 (4)	−0.022 (4)	0.012 (3)	0.058 (4)
O5	0.059 (3)	0.131 (5)	0.078 (4)	0.022 (3)	0.010 (3)	−0.017 (3)

### Geometric parameters (Å, °)

**Table:**

Ag1—N2	2.287 (5)	C6—C7	1.487 (8)
Ag1—O3	2.442 (6)	C7—C8	1.377 (10)
Ag1—N1	2.442 (6)	C7—C11	1.397 (9)
Ag1—O1^i^	2.470 (5)	C8—C9	1.372 (10)
C1—N2	1.354 (10)	C8—H8	0.9300
C1—C2	1.376 (10)	C9—C10	1.376 (10)
C1—H1	0.9300	C9—H9	0.9300
C2—C3	1.372 (10)	C10—N1	1.326 (9)
C2—H2	0.9300	C10—H10	0.9300
C3—C4	1.377 (9)	C11—N1	1.341 (7)
C3—H3	0.9300	C11—C12	1.474 (10)
C4—C12	1.405 (8)	C12—N2	1.340 (8)
C4—C5	1.486 (9)	N3—O3	1.195 (8)
C5—O2	1.218 (7)	N3—O5	1.210 (9)
C5—C6	1.499 (7)	N3—O4	1.222 (9)
C6—O1	1.211 (7)	O1—Ag1^ii^	2.470 (5)
			
N2—Ag1—O3	120.4 (2)	C9—C8—C7	118.7 (7)
N2—Ag1—N1	70.10 (19)	C9—C8—H8	120.6
O3—Ag1—N1	92.7 (2)	C7—C8—H8	120.6
N2—Ag1—O1^i^	129.06 (19)	C8—C9—C10	118.8 (7)
O3—Ag1—O1^i^	101.00 (18)	C8—C9—H9	120.6
N1—Ag1—O1^i^	140.18 (19)	C10—C9—H9	120.6
N2—C1—C2	122.8 (7)	N1—C10—C9	123.5 (6)
N2—C1—H1	118.6	N1—C10—H10	118.2
C2—C1—H1	118.6	C9—C10—H10	118.2
C1—C2—C3	119.2 (7)	N1—C11—C7	121.5 (6)
C1—C2—H2	120.4	N1—C11—C12	117.2 (6)
C3—C2—H2	120.4	C7—C11—C12	121.3 (6)
C2—C3—C4	118.9 (7)	N2—C12—C4	121.1 (6)
C2—C3—H3	120.6	N2—C12—C11	118.1 (5)
C4—C3—H3	120.6	C4—C12—C11	120.8 (6)
C3—C4—C12	119.7 (7)	C10—N1—C11	118.2 (6)
C3—C4—C5	120.1 (6)	C10—N1—Ag1	127.0 (4)
C12—C4—C5	120.2 (6)	C11—N1—Ag1	114.7 (4)
O2—C5—C4	120.9 (6)	C12—N2—C1	118.3 (6)
O2—C5—C6	120.0 (6)	C12—N2—Ag1	119.6 (4)
C4—C5—C6	119.1 (5)	C1—N2—Ag1	122.0 (4)
O1—C6—C7	122.1 (6)	O3—N3—O5	124.7 (9)
O1—C6—C5	119.9 (6)	O3—N3—O4	119.6 (9)
C7—C6—C5	118.0 (6)	O5—N3—O4	115.7 (8)
C8—C7—C11	119.3 (6)	C6—O1—Ag1^ii^	113.8 (4)
C8—C7—C6	120.0 (6)	N3—O3—Ag1	120.0 (6)
C11—C7—C6	120.7 (6)		

Symmetry codes: (i) *x*−1/2, −*y*+1/2, *z*+1/2; (ii) *x*+1/2, −*y*+1/2, *z*−1/2.

### Hydrogen-bond geometry (Å, °)

**Table:**

*D*—H···*A*	*D*—H	H···*A*	*D*···*A*	*D*—H···*A*
C1—H1···O4^iii^	0.93	2.37	3.21 (1)	149.

Symmetry codes: (iii) −*x*, −*y*, −*z*+1.
